# Impact of Hepatitis B Virus Coinfection on Human T-Lymphotropic Virus Type 1 Clonality in an Indigenous Population of Central Australia

**DOI:** 10.1093/infdis/jiy546

**Published:** 2018-10-11

**Authors:** Jocelyn Turpin, David Yurick, Georges Khoury, Hai Pham, Stephen Locarnini, Anat Melamed, Aviva Witkover, Kim Wilson, Damian Purcell, Charles R M Bangham, Lloyd Einsiedel

**Affiliations:** 1 Section of Virology, Division of Infectious Diseases, Imperial College, London, United Kingdom; 2 Department of Microbiology and Immunology, University of Melbourne, Peter Doherty Institute for Infection and Immunity, Melbourne, Victoria; 3 Baker Heart and Diabetes Institute Central Australia, Alice Springs Hospital, Northern Territory, Melbourne, Victoria, Australia; 4 Victorian Infectious Diseases Reference Laboratory, Doherty Institute, Melbourne, Victoria, Australia; 5 National Serological Reference Laboratory, Melbourne, Victoria, Australia

**Keywords:** HTLV-1/HBV coinfection, clonal expansion, Indigenous Australian population, oligoclonality

## Abstract

The prevalence of human T-cell lymphotropic virus type 1 (HTLV-1) and hepatitis B virus (HBV) coinfection is high in certain Indigenous Australian populations, but its impact on HTLV-1 has not been described. We compared 2 groups of Indigenous adults infected with HTLV-1, either alone or coinfected with HBV. The 2 groups had a similar HTLV-1 proviral load, but there was a significant increase in clonal expansion of HTLV-1–infected lymphocytes in coinfected asymptomatic individuals. The degree of clonal expansion was correlated with the titer of HBV surface antigen. We conclude that HTLV-1/HBV coinfection may predispose to HTLV-1–associated malignant disease.

## Teaser

Human T-cell lymphotropic virus type 1 (HTLV-1) and hepatitis B virus coinfection is frequent in certain Indigenous Australian populations, but its outcome remains unknown. We report a higher degree of HTLV-1 clonal expansion than in those with HTLV-1 alone.

Human T-cell lymphotropic virus type 1 (HTLV-1) and hepatitis B virus (HBV) are 2 oncogenic viruses endemic among Indigenous Australians in Central Australia [1, 2]. HTLV-1 was first detected in Central Australia in 1988, but has been intensively studied in the Indigenous population only in the last 10 years. There is no public health strategy in Australia to control HTLV-1 transmission, although the infection is highly prevalent in certain Indigenous groups: HTLV-1 seropositivity was 33.3% in a large hospital-based cohort [1], and 30 of 74 adults tested were HTLV-1 infected in a remote Indigenous community [3]. HTLV-1 imposes a significant health burden on these populations [1, 3, 4]. HBV also remains widespread in the Indigenous population of Central Australia, with an estimated prevalence of 3.96% [2]. The rate of coinfection with HTLV-1 and HBV is high in Indigenous adults in this region: HBV infection was detected in 19.2% and 15.9% of HTLV-1–seropositive Indigenous adults admitted to Alice Springs Hospital (ASH) in Central Australia [1] and patients in Northern Australia [5], respectively. Coinfection with HTLV-1 and HBV has previously been described in South America [6] and southern Japan [7]. An 8-year retrospective study of patients in Northern Australia suggested that HTLV-1–infected individuals were less able to clear HBV [5], but the impact of this coinfection on HTLV-1 has not been described. Patients with other chronic coinfections associated with HTLV-1, namely infective dermatitis and *Strongyloides stercoralis*, control the clonal abundance of HTLV-1–infected T cells less efficiently than non-coinfected individuals [8]. The aim of this study was to quantify the impact of HBV coinfection on the clonality—the clone frequency distribution—of HTLV-1–infected T cells.

## METHODS

### Ethics Approval

The study was approved by the Central Australian Human Research Ethics Committee (HREC-14–217). All patients gave informed consent in their primary languages.

### HTLV-1 and HBV Serological Studies

Plasma, peripheral blood buffy coat cells [4], or peripheral blood mononuclear cells, obtained by density-gradient centrifugation, were stored at –80°C and in liquid nitrogen until they could be shipped for HBV and HTLV-1 studies, respectively. HTLV-1 serological status was determined using an enzyme immunoassay (Murex HTLV-I + II, DiaSorin, Italy) and a particle agglutination assay (Serodia HTLV-1, Fujirebio, Tokyo, Japan). Any sample positive for one or both of these assays was validated by Western blotting (HTLV-I/II Blot2.4, MP Biomedicals Asia Pacific Pte Ltd, Singapore).

HBV viral load and hepatitis B quantitative serology for the hepatitis B surface antigen (HBsAg) were processed by the Victoria Infectious Diseases Reference Laboratory, Melbourne, Australia.

*Strongyloides* serology status was assessed by an in-house enzyme-linked immunosorbent assay.

### DNA Extraction

Genomic DNA (gDNA) was extracted from peripheral blood buffy coats [1] or from purified peripheral blood mononuclear cells, using the Qiagen DNA extraction kits (DNeasy Blood and Tissue Kit, QIA blood Mini Extraction) or the GenElute Blood Genomic DNA Kit (Sigma-Aldrich), according to the manufacturers’ instructions.

### Quantification of Proviral Load

The HTLV-1 proviral load was measured as previously described [9] by droplet digital polymerase chain reaction (ddPCR) using 100 ng of gDNA mixed with forward (5ʹ-TCCAGGCCTTATTTGGACAT-3ʹ) and reverse (5ʹ-CGTGTGAGAGTAGGACTGAG-3ʹ) primers (each 900 nM) targeting HTLV-1 *tax,* and TaqMan MGB probe (5ʹ-FAM-CATGATTTCCGGGCCTTGC-MGBNFQ-3ʹ) (250 nM) (Thermo Scientific), and (×20) PrimePCR ddPCR copy number assay: ribonuclease P/MRP 30 kDa subunit (RPP30-HEX) human primer/probe mix (Bio-Rad), and (×2) Bio-Rad Supermix for Probes (no deoxyuridine triphosphate).

All samples were run in duplicate, and the reported HTLV-1 subtype c (HTLV-1c) proviral load is the mean of the 2 measurements. The T-cell population was determined by measuring the loss of the Dβ1-Jβ1.1 intergenic sequences, as previously described [9].

The HTLV-1 proviral load was determined as the mean number of *tax* copies per 10^6^ T cells. The ddPCR limit of detection for HTLV-1 proviral load was determined to be 98 copies per 10^6^ T cells.

### Integration Site Analysis

The 5ʹ long terminal repeat (LTR) of the HTLV-1 provirus was amplified using primers targeting the end of the 5ʹ LTR (RU5) and the beginning of the *gag* open reading frame, adapted to HTLV-1c, 5LTRFW1c: 5ʹ- CTCGCTTCTTTCCCTCACG-3ʹ, 5LTRRV1c: 5ʹ-GCGCTACGAGGGAAGATTTG-3ʹ. Amplified LTR products were Sanger sequenced (accession numbers: MG783271–MG783311) and the sequences were used to design primers for ligation-mediated polymerase chain reaction (LM-PCR) and sequencing: these primers were specific for HTLV-1c sequences (Supplementary Table 1) based on previously published primers [10]. Apart from the use of these new primers, LM-PCR was performed as described previously [8]. Libraries were mixed and sequenced on 2 HiSeq 2500, rapid flow cells (2 lanes, 50 paired-end, Illumina) with samples control common to the 2 flow cells to guarantee similar sequencing efficiency. The reads were aligned to the human genome reference (UCSC hg18, excluding haplotype and “random” sequences) and the HTLV-1 reference. The only difference was the use of HTLV-1c reference for the alignment of the reads (accession number: KF242506). The quantification of number of copies of each unique integration site was deduced from the respective number of shear sites and corrected using a calibration curve, as previously described [8]. See Supplementary Tables 2 and 3 for details of sequencing results.

### Statistical Analyses

Statistical analysis was carried out using R version 3.4.1 (http://www.R-project.org/). As previously described, the oligoclonality index (an application of the Gini index) is a measure of the frequency dispersion of a cell population, and ranges from zero to 1 [8]. An oligoclonality index = 0 reflects a polyclonal cell population in which all the clones have the same frequency, and an oligoclonality index = 1 corresponds to a monoclonal population in which a single infected clone carries all the proviral load. The oligoclonality index was calculated using the ineq package (https://cran.rproject.org/web/packages/ineq/index.html) on samples with >5 unique integration sites, and adjusted for type I error [4] to limit underestimation of the oligoclonality index due to a small number of detected clones. The Mann–Whitney/Wilcoxon rank-sum test and the Spearman correlation (2 nonparametric tests) were carried out in R using the functions wilcox.test (with paired = FALSE) and cor.test (with method = “spearman”), respectively.

## RESULTS

All Indigenous adults (>18 years old) presenting to ASH and found to be HBsAg positive were eligible for recruitment. ASH is the only medical facility serving a 1 × 10^6^ km^2^ area in Central Australia. Viral hepatitis at ASH is managed largely via telehealth; few subjects attend clinic in person. Subjects were recruited from April 2014 to December 2015 inclusive, and offered tests for HTLV-1 infection and HBV infection. Consent was obtained in all cases in the subject’s primary language by Indigenous research staff who were unaware of the clinical details.

**Figure 1.**
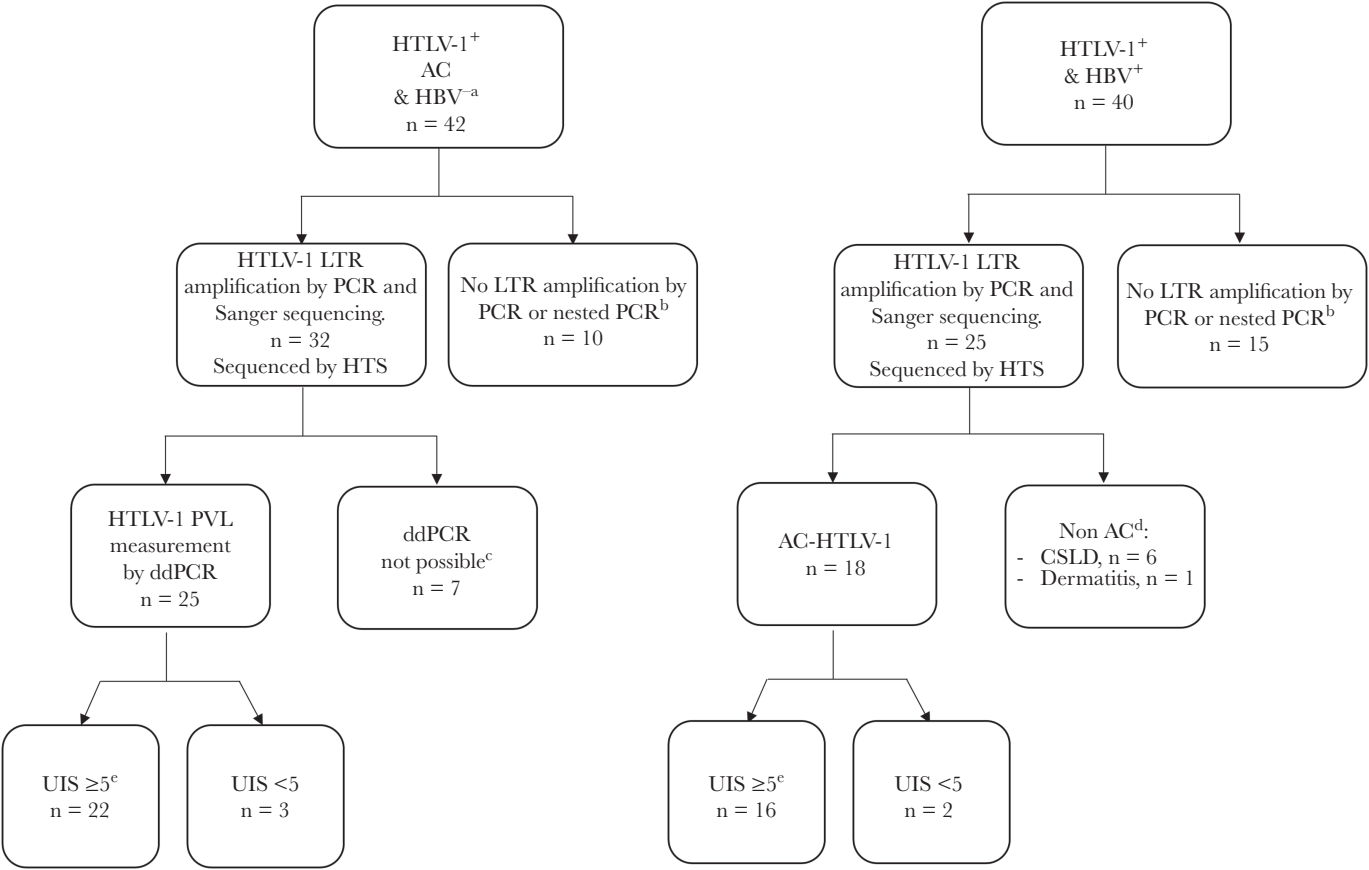
Selection of human T-cell lymphotropic virus type 1 (HTLV-1)–monoinfected subjects and individuals coinfected with hepatitis B virus (HBV) for the clonality study. ^a^The group of asymptomatic monoinfected subjects was initially described in [4] and used as a control to compare the clonality of HTLV-1 between asymptomatic and symptomatic subjects (chronic suppurative lung disease and uveitis). ^b^Of 42 monoinfected samples, 10 yielded no polymerase chain reaction (PCR) product. Three of these 10 samples were sequenced, but no usable sequences were obtained. These 10 samples were therefore not analyzed. Similarly, of 40 coinfected samples, 15 yielded no PCR product. Four of these 15 samples were sequenced, but no usable sequences were obtained. These 15 samples were therefore not analyzed further. ^c^To compare the 2 groups, the HTLV-1 proviral load of T cells was remeasured by droplet digital PCR ([9]). Sufficient DNA was not available for 7 samples from the previous study [4]. ^d^Only asymptomatic carriers were included in the clonality analysis. Six coinfected patients with chronic suppurative lung disease and one with infective dermatitis were removed. ^e^Three monoinfected and 2 coinfected samples were discarded because the small number of unique integration sites precluded an accurate estimate of the oligoclonality index in these cases. None of the remaining 16 patients was receiving antiviral therapy for HBV; however, every isolate in this population to date belongs to the HBV subgenotype C4, as previously described [15]. Abbreviations: AC, asymptomatic human T-cell lymphotropic virus type 1 carrier; CSLD, chronic suppurative lung disease; ddPCR, droplet digital polymerase chain reaction; HBV, hepatitis B virus; HTLV-1, human T-cell lymphotropic virus type 1; HTS, high-throughput sequencing; LTR, long terminal repeat; PCR, polymerase chain reaction; PVL, proviral load; UIS, unique integration sites.

HTLV-1 parameters for HBV/HTLV-1–coinfected subjects were compared with those of asymptomatic HTLV-1–monoinfected subjects recruited from December 2008 to November 2013 inclusive [4]; the selection of reported patients is described in Figure 1.

**Figure 2.**
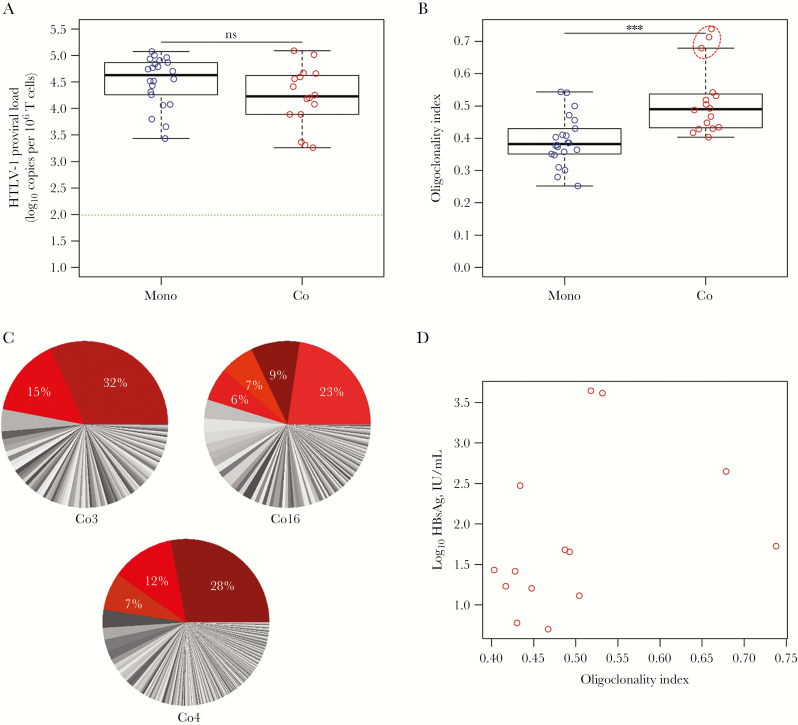
Individuals coinfected with human T-cell lymphotropic virus type 1 (HTLV-1) and hepatitis B virus (HBV) (n = 16) had a higher oligoclonality index than HTLV-1–monoinfected individuals (n = 22) in T cells. *A*, HTLV-1 proviral load was measured in duplicate by droplet digital polymerase chain reaction, using a *tax* probe as a viral specific target and RPP30 as an internal reference gene. The T-cell count was then determined by estimating the frequency of non–T cells by using a set of primers targeting the unrearranged T-cell receptor Dβ1-Jβ1.1 intergenic sequences [9]. The limit of detection of 98 copies per million T cells is indicated by the dashed lines. The proviral load in T cells was not significantly different between the 2 groups (the median proviral load of monoinfected and coinfected individuals was 4.3 × 10^4^ and 1.7 × 10^4^ proviral copies per million T cells, respectively; Wilcoxon rank-sum test, *P* = .10). *B*, The oligoclonality distribution of coinfected subjects measured by the oligoclonality index (median oligoclonality index of 0.490) was significantly higher than the oligoclonality index of monoinfected subjects (median oligoclonality index = 0.382; Wilcoxon rank-sum test, *P* = .0003). *C*, Clone frequency distribution of the 3 coinfected patients within the red dashed oval in (*B*). Each slice of the pie chart corresponds to a unique integration site and its width is directly proportional to its relative abundance. The frequency of the most abundant clones is indicated in the corresponding slice. These expanded clones carry a significant fraction of the proviral load in this individual, whose oligoclonality index lies within the range typical of adult T-cell leukemia [10, 12]. *D*, The titer of HBV surface antigen correlated positively with oligoclonality index (*P* = .03, *R* = 0.56, Spearman). ***P < .001; not significant, *P* > .05. Abbreviations: Co, coinfected; HBsAg, hepatitis B surface antigen; HTLV-1, human T-cell lymphotropic virus type 1; Mono, monoinfected; ns, not significant.

Genomic DNA was extracted from peripheral blood buffy coats for the initial study in asymptomatic HTLV-1–monoinfected subjects [4] and from peripheral blood mononuclear cells in the coinfection study. To compare the proviral load of HTLV-1 between these 2 different sets of samples, we quantified the proviral load in T cells by a ddPCR assay [9]. The proviral load in HTLV-1–infected subjects coinfected with HBV (median, 1.7 × 10^4^ proviral copies per 10^6^ T cells) was not significantly different from that of HTLV-1–monoinfected asymptomatic subjects (median, 4.3 × 10^4^ proviral copies per 10^6^ T cells; Figure 2A; Wilcoxon rank-sum test, *P* = .10). The degree of oligoclonal expansion of infected lymphocytes is measured by the oligoclonality index [10]. The oligoclonality index in coinfected subjects (median, 0.490) was significantly greater than that in HTLV-1–monoinfected individuals (median, 0.382; Figure 2B, Wilcoxon rank-sum test, *P* = .0003). Three patients (Co3, Co16, Co4; Supplementary Table 2) had high oligoclonality index values (0.679, 0.718, and 0.738, respectively), which are in the lower end of the range typical of adult T-cell leukemia patients (Figure 2B). In each of these individuals, 2 to 4 clones (Figure 2C) accounted for almost half of the proviral load (Supplementary Table 3).

There was no correlation between the HBV load and either the oligoclonality index or the proviral load of HTLV-1 (Supplementary Table 2). The titer of HBsAg (quantitative HBsAg [qHBsAg] assay), available for all coinfected patients except one (Co16), was positively correlated with the HTLV-1 oligoclonality index (Figure 2D; *P* = .03, *R* = 0.56, Spearman), but was not correlated with the HTLV-1 proviral load (Supplementary Table 2). All patients had negative (n = 37) or equivocal (n = 3) *S. stercoralis* serological status (Supplementary Table 2) except one patient (Co5) in whom serological status was not available but who had no clinical evidence of active strongyloidiasis. However, the difference in oligoclonality index between the monoinfected and coinfected groups (*P* = .0005) remained significant even when this patient was omitted. Nonetheless the correlation between qHBsAg titers and HTLV-1 oligoclonality index remained marginally significant (*P* = .05, *R* = 0.53, Spearman).

## DISCUSSION

Contrary to *Strongyloides*–HTLV-1 coinfection and subjects with infective dermatitis [8, 11], HBV coinfection had no impact in this study on the HTLV-1 proviral load. However, the greater oligoclonality observed in the coinfected patients resembles the HTLV-1 clonality previously reported in HTLV-1 coinfection with *S. stercoralis* [8, 11] and suggests that these individuals are less able than monoinfected individuals to restrict the expansion of certain subsets of HTLV-1–infected lymphocytes. Our results therefore indicate that coinfection with HBV may increase the risk of HTLV-1–induced malignant disease, because a high oligoclonality index is known to be associated with adult T-cell leukemia [10, 12.There was no significant correlation between the oligoclonality index and the proviral load in either group (Supplementary Table 2), as previously found in other populations in nonmalignant HTLV-1 infection [10] and in patients with infective dermatitis or coinfected with *S. stercoralis* [8].The positive correlation observed between the oligoclonality index and the qHBsAg titer suggests that HBV infection drives the HTLV-1 clonal expansion observed in coinfected patients, perhaps by persistent antigenic stimulation of HTLV-1–infected T-cell clones specific to the coinfecting pathogen, as previously suggested in coinfection with HTLV-1 and *Strongyloides* [8].

Although previous work did not indicate an association between HBsAg positivity and the presence of HTLV-1 infection in a multivariate model [1], it will be important to ascertain the impact of HBV coinfection on HTLV-1–associated diseases with longitudinal studies.

In the present study, the coinfected group had a higher median age than monoinfected subjects (Supplementary Figure 1 and Supplementary Table 2). However, the rate of increase of oligoclonality index with age [10] is too low to explain the observed difference in mean oligoclonality index between the 2 groups in this study. The age difference between the 2 groups might be explained by an increase in the probability of coinfection with another chronic infection with age; to be tested, this hypothesis would require more patients to be recruited.

Also, there was a wide variation in proviral load (up to 68-fold) within each group, as reported previously in both asymptomatic carriers and patients with inflammatory disease. Our results raise the question whether the impact of HBV coinfection on T-cell clonality is to alter the nature or magnitude of specific T-cell populations, for example, by stimulating T-cell proliferation, expression of CD25 (interleukin 2 receptor alpha chain [13]), or the frequency of CD4^+^FoxP3^+^ regulatory T cells [14].

In this study we quantified the impact of coinfection with HBV on HTLV-1 clonality and proviral load. Serological and epidemiological studies also suggest that the coinfection may limit the control of HBV infection [5]. Finally, the unique HBV subgenotype C4 infecting Indigenous Australians in Central and Northern Australia carries mutations associated with both faster liver disease progression and a higher risk of hepatocellular carcinoma [15]; longitudinal studies and further molecular and cellular investigations are needed to quantify the impact of the coinfection on the risk of HBV-associated diseases.

## Supplementary Data

Supplementary materials are available at *The Journal of Infectious Diseases* online (http://jid.oxfordjournals.org/). Supplementary materials consist of data provided by the author that are published to benefit the reader. The posted materials are not copyedited. The contents of all supplementary data are the sole responsibility of the authors. Questions or messages regarding errors should be addressed to the author.

## Supplementary Material

Supplemental_Figure and Table-1

Supplemental_Table_2

Supplemental_Table_3

## Notes

***Acknowledgments.*** We are grateful to Laurence Game and Marian Dore of the Genomics Laboratory, Medical Research Council Clinical Sciences Centre, Hammersmith Hospital, London, United Kingdom, for their assistance with sequencing.

***Financial support.*** This work was supported by the Australian Centre for HIV and Hepatitis Virology Research, which is funded by the Department of Health and Ageing, Australian Commonwealth Government; and by the Wellcome Trust (Charles Bangham Senior Investigator Award number WT100291MA).

***Potential conflicts of interest.*** All authors: No reported conflicts of interest. All authors have submitted the ICMJE Form for Disclosure of Potential Conflicts of Interest. Conflicts that the editors consider relevant to the content of the manuscript have been disclosed.
