# Supplementary material for: Impact of Hepatitis B Virus Coinfection on Human T-Lymphotropic Virus Type 1 Clonality in an Indigenous Population of Central Australia
Source: J Infect Dis. 2018 Oct 11;219(4):562–7. doi: 10.1093/infdis/jiy546 (PMC6350946; doi:10.1093/infdis/jiy546)
Supplement: Supplemental_Figure and Table-1 [file jiy546_suppl_supplemental_figure-table-1.docx]

**Supplemental Data for:**

**Impact of Hepatitis B Virus co-infection on Human T-Lymphotropic Virus type 1 clonality in an indigenous population of Central Australia.**

Turpin et al

Published in: Journal of Infectious Diseases


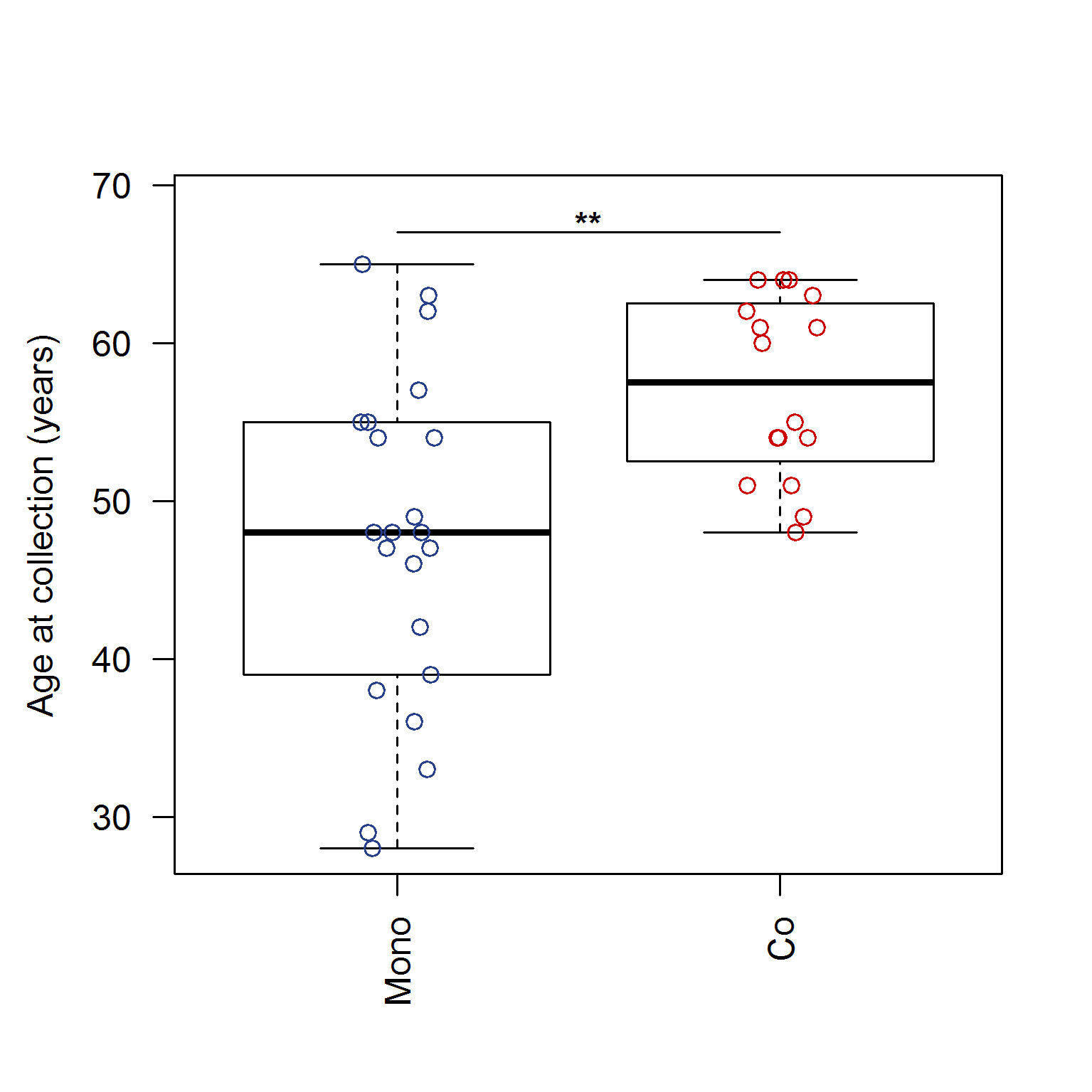


Supplemental figure 1: The age of the mono-infected cohort was statistically significantly lower than the age of the co-infected cohort, with median ages of respectively 48 and 57.5 years (Wilcoxon rank sum test, p = 0.004).

| Name | Target | Step | Sequence (5' 🡪 3') |
| --- | --- | --- | --- |
| Bio3-f | LTR | LM-PCR | CCTTTCATTCACGTCTGACTGCCG |
| P5-Bio5 | LTR |  | AATGATACGGCGACCACCGAGATCTACACTGGCTCGGAGCCAGCGACAGCCCAT |
| Bio4 | Linker |  | TCATGATCAATGGGACGATCA |
| P7 | Linker |  | CAAGCAGAAGACGGCATACGA |
| H1.CN | LTR | Sequencing Primers : Read 1 | CAGCCCATTCTATACCTCTCTCCAGGAGAGAGACATAGA |
| H1.CS | LTR | Sequencing Primers : Read 1 | CAGCCCATTCTATACCTCTCTCCAGGAGAGAGACTTAGA |
| SBS8rev | Linker | Sequencing Primers : Index read | AGATCGGAAGAGCGGTTCAGCAGGAATGCCGAGACCG |
| SBS8+T | Linker | Sequencing Primers : Read 2 | CGGTCTCGGCATTCCTGCTGAACCGCTCTTCCGATCT |

Supplemental table 1: Primers used for the linker-mediated PCR and the high-throughput sequencing.

Supplemental table 2**:** Characteristics of persons positive for HTLV-1c only or co-infected by HBV and HTLV-1c.

Supplemental table 3: Identified integration sites.
